# {5,10,15,20-Tetra­kis[4-(oct­yloxy)phen­yl]porphyrinato}copper(II)

**DOI:** 10.1107/S1600536811043698

**Published:** 2011-11-05

**Authors:** De-Liang Yang, Hong-Bin Zhao, Jun-Xu Liao, Liang Chen, Bang-Ying Wang

**Affiliations:** a Department of Organic Chemistry, The College of Chemistry, Xiangtan University, Hunan 411105, People’s Republic of China; b College of Chemistry and Environmental Engineering, Dongguan University of Technology, Guangdong 523808, People’s Republic of China; c Cleaner Production Center, Dongguan University of Technology, Guangdong 523808, People’s Republic of China

## Abstract

In the title compound, [Cu(C_76_H_92_N_4_O_4_)], the central Cu(II) ion is situated on an inversion centre. The porphyrinate core exhibits a nearly planar conformation [maximum deviation = 0.027 (3) Å], with Cu—N distances of 1.997 (2) and 2.001 (2) Å. The benzene rings of the 4-octyloxyphenyl groups are rotated at angles of 84.02 (8) and 77.02 (6)° with respect to the mean plane of the porphyrin fragment. The two terminal C atoms in the octyl group are disordered over two positions of equal occupancy.

## Related literature

For general background to porphyrin species and their applications, see: Holten *et al.* (2002 ▶); Gust & Moore (1985 ▶); Gunter & Johnston (1992 ▶); Anderson & Sanders (1995 ▶). For related structures, see: Fleischer (1963 ▶); Fleischer *et al.* (1964 ▶); He (2007 ▶).
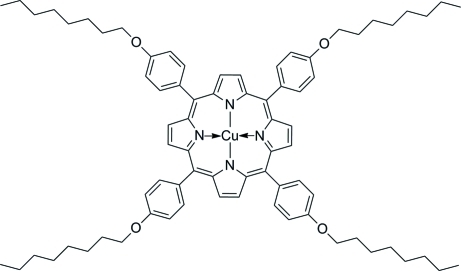


## Experimental

### 

#### Crystal data

[Cu(C_76_H_92_N_4_O_4_)]
*M* *_r_* = 1189.08
Monoclinic, 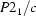

*a* = 16.0521 (16) Å
*b* = 19.2628 (18) Å
*c* = 10.4767 (10) Å
β = 90.024 (2)°
*V* = 3239.5 (5) Å^3^
*Z* = 2
Mo *K*α radiation
μ = 0.39 mm^−1^
*T* = 185 K
0.22 × 0.14 × 0.08 mm

#### Data collection

Bruker APEX CCD area-detector diffractometer
Absorption correction: multi-scan (*SADABS*; Sheldrick, 2004 ▶) *T*_min_ = 0.919, *T*_max_ = 0.970
17425 measured reflections
5732 independent reflections
3710 reflections with *I* > 2σ(*I*)
*R*_int_ = 0.064

#### Refinement

*R*[*F* ^2^ > 2σ(*F* ^2^)] = 0.050
*wR*(*F* ^2^) = 0.124
*S* = 1.03
5732 reflections
406 parameters
4 restraints
H-atom parameters constrained
Δρ_max_ = 0.52 e Å^−3^
Δρ_min_ = −0.43 e Å^−3^

### 

Data collection: *SMART* (Bruker, 2002 ▶); cell refinement: *SAINT* (Bruker, 2002 ▶); data reduction: *SAINT*; program(s) used to solve structure: *SHELXS97* (Sheldrick, 2008 ▶); program(s) used to refine structure: *SHELXL97* (Sheldrick, 2008 ▶); molecular graphics: *SHELXTL* (Sheldrick, 2008 ▶); software used to prepare material for publication: *SHELXTL*.

## Supplementary Material

Crystal structure: contains datablock(s) global, I. DOI: 10.1107/S1600536811043698/lx2202sup1.cif

Structure factors: contains datablock(s) I. DOI: 10.1107/S1600536811043698/lx2202Isup2.hkl

Additional supplementary materials:  crystallographic information; 3D view; checkCIF report

## Figures and Tables

**Fig. 1.**
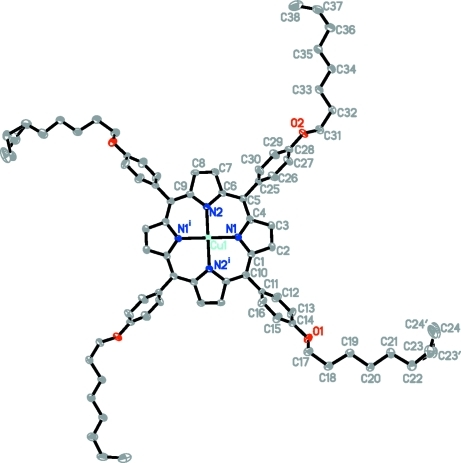
The molecular structure of the title compound with the atom numbering scheme. Displacement ellipsoids are drawn at the 50% probability level. Two terminal C atoms (C23 & C24) in the octyl group are disordered over two positions with site occupancy factors, from refinement of 0.4510 (7) (part A) and 0.5489 (3) (part B). H atoms were omitted for clarity. [Symmetry codes: (i) - x + 1, - y + 1, - z + 1.]

## supplementary crystallographic information

### Comment

Porphyrins, metalloporphyrins, and their derivatives are researched in many fields, such as molecular electronic devices (Holten *et al.*, 2002), natural photosynthetic systems (Gust & Moore, 1985), electronic devices (Gunter & Johnston, 1992) or enzyme mimics (Anderson & Sanders, 1995). In this paper, we report the crystal structure of the title compound.

The porphyrin moiety in the title compound is essentially planar, the macrocyclic core 24-menbered ring is planar with the mean deviation of 0.027 (3) Å. The fourcoordinate Cu ion fitting into its center at 1.997 (2)-2.001 (2) Å, from the surrouding pyrrole N atoms, in agreement with that found in the related compounds (Fleischer 1963; Fleischer *et al.*, 1964; He 2007.). The *p*-octyloxyphenyl groups are rotated at angles of 84.02 (8) ° (C11-C16) and 77.02 (6) ° (C25-C30) with respect to the porphyrin mean plane, due to steric hindrance with the pyrrole-H atoms of the macrocycle. Two terminal C atoms (C23 & C24) in the octyl group are disordered over two positions with site occupancy factors, from refinement of  0.4510 (7) (part A) and 0.5489 (3) (part B).

### Experimental

0.04mmol meso-tetrakis[p-(octyloxy)phenyl] porphyrin and 0.40mmol Cu(CH_3_COO)_2_.H_2_O were dissolved in 20 ml chloroform, refluxed for 8 hours, and the solvent was removed by a rotary evaporator, the residue was purified by column chromatography with chloroform, then crystallized by methanol and chloroform, and a purple solid was obtained (yield=55%). Single crystals were recrystallization from a dichloromethane solution at room temperature.

### Refinement

H atoms were positioned geometrically and refined using a riding model, with C–H = 0.95 (aromatic), 0.99 (CH_2_) and 0.98 (CH_3_) Å and with *U*_iso_(H) = 1.2(1.5 for methyl)*U*_eq_(C). Two terminal C atoms (C23 & C24) in the octyl group are disordered over two positions with site occupancy factors, from refinement of 0.4510 (7) (part A) and 0.5489 (3) (part B). The distance of equivalent C-C pairs were restrained to 1.540 (1) Å and 0.001 Å using command DFIX and SADI, respectively, and displacement ellipsoids of C23 & C24 set were restrained to 0.01 using command ISOR and DELU.

### Crystal data

**Table:**

[Cu(C_76_H_92_N_4_O_4_)]	*F*(000) = 1274
*M**_r_* = 1189.08	*D*_x_ = 1.219 Mg m^−^^3^
Monoclinic, *P*2_1_/*c*	Mo *K*α radiation, λ = 0.71073 Å
Hall symbol: -P 2ybc	Cell parameters from 3031 reflections
*a* = 16.0521 (16) Å	θ = 2.5–23.5°
*b* = 19.2628 (18) Å	µ = 0.39 mm^−^^1^
*c* = 10.4767 (10) Å	*T* = 185 K
β = 90.024 (2)°	Block, purple
*V* = 3239.5 (5) Å^3^	0.22 × 0.14 × 0.08 mm
*Z* = 2	

### Data collection

**Table:**

Bruker APEX CCD area-detector diffractometer	5732 independent reflections
Radiation source: fine-focus sealed tube	3710 reflections with *I* > 2σ(*I*)
graphite	*R*_int_ = 0.064
phi and ω scans	θ_max_ = 25.1°, θ_min_ = 1.7°
Absorption correction: multi-scan (*SADABS*; Sheldrick, 2004)	*h* = −16→19
*T*_min_ = 0.919, *T*_max_ = 0.970	*k* = −22→22
17425 measured reflections	*l* = −12→11

### Refinement

**Table:**

Refinement on *F*^2^	Primary atom site location: structure-invariant direct methods
Least-squares matrix: full	Secondary atom site location: difference Fourier map
*R*[*F*^2^ > 2σ(*F*^2^)] = 0.050	Hydrogen site location: difference Fourier map
*wR*(*F*^2^) = 0.124	H-atom parameters constrained
*S* = 1.03	*w* = 1/[σ^2^(*F*_o_^2^) + (0.0573*P*)^2^ + 0.5451*P*] where *P* = (*F*_o_^2^ + 2*F*_c_^2^)/3
5732 reflections	(Δ/σ)_max_ = 0.002
406 parameters	Δρ_max_ = 0.52 e Å^−^^3^
4 restraints	Δρ_min_ = −0.43 e Å^−^^3^

### Special details

**Table:**

Geometry. All e.s.d.'s (except the e.s.d. in the dihedral angle between two l.s. planes) are estimated using the full covariance matrix. The cell e.s.d.'s are taken into account individually in the estimation of e.s.d.'s in distances, angles and torsion angles; correlations between e.s.d.'s in cell parameters are only used when they are defined by crystal symmetry. An approximate (isotropic) treatment of cell e.s.d.'s is used for estimating e.s.d.'s involving l.s. planes.
Refinement. Refinement of *F*^2^ against ALL reflections. The weighted *R*-factor *wR* and goodness of fit *S* are based on *F*^2^, conventional *R*-factors *R* are based on *F*, with *F* set to zero for negative *F*^2^. The threshold expression of *F*^2^ > σ(*F*^2^) is used only for calculating *R*-factors(gt) *etc*. and is not relevant to the choice of reflections for refinement. *R*-factors based on *F*^2^ are statistically about twice as large as those based on *F*, and *R*- factors based on ALL data will be even larger.

### Fractional atomic coordinates and isotropic or equivalent isotropic displacement parameters (Å^2^)

**Table:**

	*x*	*y*	*z*	*U*_iso_*/*U*_eq_	Occ. (<1)
Cu1	0.0000	0.0000	0.0000	0.01922 (16)	
N1	0.06186 (15)	0.08619 (10)	0.0474 (2)	0.0187 (6)	
N2	−0.09244 (15)	0.05769 (11)	−0.0721 (2)	0.0210 (6)	
O1	0.47610 (14)	0.09309 (10)	0.4409 (2)	0.0348 (6)	
O2	−0.09354 (13)	0.46495 (9)	−0.07939 (19)	0.0286 (5)	
C1	0.13671 (19)	0.09088 (14)	0.1114 (3)	0.0210 (7)	
C2	0.1588 (2)	0.16259 (14)	0.1298 (3)	0.0248 (8)	
H2	0.2070	0.1797	0.1721	0.030*	
C3	0.09840 (19)	0.20081 (14)	0.0755 (3)	0.0248 (7)	
H3	0.0967	0.2500	0.0710	0.030*	
C4	0.03719 (19)	0.15420 (14)	0.0259 (3)	0.0207 (7)	
C5	−0.03763 (19)	0.17514 (14)	−0.0293 (3)	0.0207 (7)	
C6	−0.09829 (19)	0.12931 (14)	−0.0732 (3)	0.0217 (7)	
C7	−0.17422 (19)	0.14997 (15)	−0.1334 (3)	0.0261 (8)	
H7	−0.1929	0.1963	−0.1458	0.031*	
C8	−0.2143 (2)	0.09192 (14)	−0.1693 (3)	0.0279 (8)	
H8	−0.2666	0.0895	−0.2115	0.033*	
C9	−0.16300 (19)	0.03415 (14)	−0.1318 (3)	0.0228 (7)	
C10	0.18378 (19)	0.03500 (14)	0.1551 (3)	0.0222 (7)	
C11	0.26082 (19)	0.05015 (13)	0.2320 (3)	0.0220 (7)	
C12	0.3363 (2)	0.06474 (16)	0.1743 (3)	0.0355 (9)	
H12	0.3399	0.0651	0.0838	0.043*	
C13	0.4071 (2)	0.07895 (16)	0.2466 (3)	0.0355 (8)	
H13	0.4584	0.0892	0.2054	0.043*	
C14	0.4027 (2)	0.07817 (14)	0.3783 (3)	0.0247 (7)	
C15	0.3287 (2)	0.06294 (16)	0.4370 (3)	0.0345 (8)	
H15	0.3257	0.0615	0.5275	0.041*	
C16	0.2579 (2)	0.04953 (16)	0.3638 (3)	0.0338 (8)	
H16	0.2066	0.0398	0.4054	0.041*	
C17	0.4775 (2)	0.08613 (16)	0.5752 (3)	0.0385 (9)	
H17A	0.4683	0.0371	0.5997	0.046*	
H17B	0.4331	0.1148	0.6142	0.046*	
C18	0.5628 (2)	0.11044 (16)	0.6209 (3)	0.0411 (10)	
H18A	0.6065	0.0836	0.5761	0.049*	
H18B	0.5681	0.1009	0.7134	0.049*	
C19	0.5769 (2)	0.18808 (15)	0.5972 (3)	0.0406 (10)	
H19A	0.5860	0.1955	0.5048	0.049*	
H19B	0.5258	0.2136	0.6216	0.049*	
C20	0.6503 (2)	0.21847 (15)	0.6702 (3)	0.0398 (9)	
H20A	0.7026	0.1981	0.6372	0.048*	
H20B	0.6454	0.2059	0.7615	0.048*	
C21	0.6541 (3)	0.29707 (17)	0.6576 (4)	0.0622 (14)	
H21A	0.5985	0.3162	0.6785	0.075*	
H21B	0.6659	0.3087	0.5673	0.075*	
C22	0.7184 (3)	0.33295 (15)	0.7409 (4)	0.0556 (12)	
H22A	0.7746	0.3170	0.7090	0.067*	0.50
H22B	0.7132	0.3124	0.8283	0.067*	0.50
H22C	0.7748	0.3157	0.7158	0.067*	0.50
H22D	0.7093	0.3198	0.8304	0.067*	0.50
C23	0.6963 (7)	0.4092 (3)	0.7736 (8)	0.058 (3)	0.50
H23A	0.7418	0.4298	0.8250	0.070*	0.50
H23B	0.6448	0.4103	0.8255	0.070*	0.50
C24	0.6835 (11)	0.4525 (9)	0.6519 (11)	0.074 (5)	0.50
H24A	0.7345	0.4516	0.6006	0.111*	0.50
H24B	0.6704	0.5005	0.6753	0.111*	0.50
H24C	0.6374	0.4329	0.6023	0.111*	0.50
C23'	0.7397 (4)	0.4077 (3)	0.6990 (8)	0.0375 (19)	0.50
H23C	0.7837	0.4274	0.7547	0.045*	0.50
H23D	0.7600	0.4081	0.6098	0.045*	0.50
C24'	0.6590 (8)	0.4505 (7)	0.7108 (15)	0.071 (4)	0.50
H24D	0.6196	0.4358	0.6446	0.106*	0.50
H24E	0.6718	0.4999	0.7001	0.106*	0.50
H24F	0.6343	0.4429	0.7952	0.106*	0.50
C25	−0.05312 (19)	0.25190 (13)	−0.0433 (3)	0.0207 (7)	
C26	−0.0115 (2)	0.28857 (15)	−0.1366 (3)	0.0331 (8)	
H26	0.0260	0.2648	−0.1914	0.040*	
C27	−0.0233 (2)	0.36000 (15)	−0.1522 (3)	0.0323 (8)	
H27	0.0057	0.3844	−0.2172	0.039*	
C28	−0.07731 (19)	0.39466 (14)	−0.0727 (3)	0.0237 (7)	
C29	−0.1196 (2)	0.35866 (15)	0.0213 (3)	0.0333 (8)	
H29	−0.1573	0.3823	0.0760	0.040*	
C30	−0.1069 (2)	0.28784 (15)	0.0352 (3)	0.0313 (8)	
H30	−0.1360	0.2635	0.1004	0.038*	
C31	−0.04455 (19)	0.50473 (14)	−0.1677 (3)	0.0291 (7)	
H31A	−0.0479	0.4840	−0.2540	0.035*	
H31B	0.0145	0.5051	−0.1406	0.035*	
C32	−0.0785 (2)	0.57783 (14)	−0.1696 (3)	0.0297 (8)	
H32A	−0.0397	0.6080	−0.2177	0.036*	
H32B	−0.0817	0.5955	−0.0811	0.036*	
C33	−0.1645 (2)	0.58193 (14)	−0.2305 (3)	0.0281 (8)	
H33A	−0.2037	0.5542	−0.1784	0.034*	
H33B	−0.1618	0.5603	−0.3161	0.034*	
C34	−0.1993 (2)	0.65486 (14)	−0.2445 (3)	0.0313 (8)	
H34A	−0.2117	0.6739	−0.1588	0.038*	
H34B	−0.1569	0.6850	−0.2848	0.038*	
C35	−0.2782 (2)	0.65601 (15)	−0.3246 (3)	0.0317 (8)	
H35A	−0.3214	0.6285	−0.2801	0.038*	
H35B	−0.2665	0.6327	−0.4069	0.038*	
C36	−0.3136 (2)	0.72779 (16)	−0.3526 (3)	0.0441 (10)	
H36A	−0.3364	0.7475	−0.2727	0.053*	
H36B	−0.2678	0.7585	−0.3815	0.053*	
C37	−0.3816 (2)	0.72724 (18)	−0.4536 (4)	0.0523 (11)	
H37A	−0.3575	0.7106	−0.5350	0.063*	
H37B	−0.4011	0.7754	−0.4672	0.063*	
C38	−0.4555 (3)	0.6823 (2)	−0.4201 (4)	0.0675 (13)	
H38A	−0.4365	0.6350	−0.4022	0.101*	
H38B	−0.4947	0.6816	−0.4918	0.101*	
H38C	−0.4833	0.7012	−0.3444	0.101*	

### Atomic displacement parameters (Å^2^)

**Table:**

	*U*^11^	*U*^22^	*U*^33^	*U*^12^	*U*^13^	*U*^23^
Cu1	0.0204 (3)	0.0137 (2)	0.0236 (3)	0.0008 (2)	−0.0081 (2)	0.0002 (2)
N1	0.0211 (15)	0.0150 (12)	0.0199 (13)	0.0008 (10)	−0.0039 (11)	−0.0011 (10)
N2	0.0232 (15)	0.0152 (12)	0.0245 (13)	0.0005 (11)	−0.0078 (12)	0.0003 (10)
O1	0.0303 (14)	0.0343 (12)	0.0398 (13)	−0.0059 (10)	−0.0178 (11)	−0.0014 (10)
O2	0.0362 (14)	0.0160 (10)	0.0338 (12)	0.0042 (9)	0.0001 (11)	0.0041 (9)
C1	0.0227 (19)	0.0195 (15)	0.0207 (16)	−0.0004 (13)	−0.0048 (14)	−0.0018 (12)
C2	0.028 (2)	0.0177 (15)	0.0288 (18)	−0.0044 (13)	−0.0066 (15)	−0.0023 (13)
C3	0.028 (2)	0.0162 (15)	0.0302 (17)	−0.0013 (13)	−0.0048 (15)	−0.0030 (13)
C4	0.027 (2)	0.0146 (14)	0.0204 (16)	−0.0006 (13)	−0.0001 (14)	−0.0010 (12)
C5	0.0239 (19)	0.0171 (15)	0.0212 (16)	0.0010 (13)	−0.0029 (14)	0.0014 (12)
C6	0.0251 (19)	0.0158 (14)	0.0241 (16)	0.0022 (13)	−0.0055 (14)	0.0010 (12)
C7	0.028 (2)	0.0173 (15)	0.0333 (18)	0.0031 (13)	−0.0088 (15)	−0.0009 (13)
C8	0.025 (2)	0.0231 (16)	0.0352 (18)	0.0033 (14)	−0.0132 (15)	0.0030 (14)
C9	0.0226 (19)	0.0210 (16)	0.0247 (17)	0.0019 (13)	−0.0059 (15)	0.0004 (13)
C10	0.0225 (19)	0.0200 (15)	0.0239 (17)	0.0004 (13)	−0.0057 (14)	−0.0013 (13)
C11	0.0231 (19)	0.0136 (13)	0.0293 (18)	0.0018 (13)	−0.0061 (15)	0.0006 (13)
C12	0.032 (2)	0.047 (2)	0.0276 (18)	−0.0055 (17)	−0.0092 (17)	0.0038 (15)
C13	0.023 (2)	0.047 (2)	0.036 (2)	−0.0092 (16)	−0.0029 (16)	0.0014 (16)
C14	0.024 (2)	0.0174 (15)	0.0324 (18)	−0.0010 (13)	−0.0105 (15)	−0.0020 (13)
C15	0.033 (2)	0.043 (2)	0.0271 (18)	−0.0059 (17)	−0.0072 (16)	−0.0046 (15)
C16	0.025 (2)	0.044 (2)	0.033 (2)	−0.0059 (16)	−0.0044 (16)	−0.0031 (15)
C17	0.037 (2)	0.0327 (18)	0.046 (2)	−0.0014 (16)	−0.0179 (18)	−0.0003 (16)
C18	0.042 (2)	0.0279 (18)	0.053 (2)	−0.0003 (16)	−0.0260 (19)	−0.0030 (16)
C19	0.041 (2)	0.0244 (17)	0.056 (2)	0.0008 (16)	−0.0239 (19)	−0.0009 (16)
C20	0.036 (2)	0.0232 (17)	0.060 (2)	−0.0016 (15)	−0.0238 (19)	−0.0013 (16)
C21	0.071 (3)	0.0264 (19)	0.089 (3)	−0.0103 (19)	−0.048 (3)	0.0037 (19)
C22	0.060 (3)	0.0280 (19)	0.079 (3)	−0.0016 (18)	−0.037 (2)	−0.0059 (19)
C23	0.067 (7)	0.038 (5)	0.071 (7)	−0.008 (5)	−0.023 (6)	−0.006 (4)
C24	0.121 (14)	0.042 (7)	0.059 (9)	−0.034 (8)	−0.011 (7)	0.003 (6)
C23'	0.036 (5)	0.031 (4)	0.046 (5)	−0.010 (3)	−0.008 (4)	−0.006 (4)
C24'	0.068 (9)	0.017 (5)	0.127 (14)	0.003 (5)	−0.013 (9)	−0.004 (9)
C25	0.0227 (18)	0.0160 (14)	0.0232 (16)	−0.0007 (13)	−0.0057 (14)	−0.0001 (13)
C26	0.044 (2)	0.0224 (16)	0.0333 (19)	0.0090 (15)	0.0062 (17)	0.0008 (14)
C27	0.042 (2)	0.0220 (16)	0.0327 (19)	0.0030 (15)	0.0068 (17)	0.0076 (14)
C28	0.027 (2)	0.0181 (14)	0.0258 (17)	0.0043 (13)	−0.0088 (15)	−0.0002 (13)
C29	0.036 (2)	0.0259 (17)	0.038 (2)	0.0071 (15)	0.0109 (17)	0.0027 (15)
C30	0.034 (2)	0.0214 (16)	0.0386 (19)	0.0001 (14)	0.0081 (17)	0.0077 (14)
C31	0.0313 (19)	0.0213 (15)	0.0346 (17)	0.0000 (15)	−0.0004 (15)	0.0068 (15)
C32	0.031 (2)	0.0189 (15)	0.0387 (19)	−0.0027 (14)	−0.0070 (16)	0.0032 (14)
C33	0.033 (2)	0.0187 (15)	0.0330 (18)	0.0009 (14)	−0.0063 (16)	0.0013 (13)
C34	0.037 (2)	0.0194 (15)	0.0372 (19)	−0.0010 (14)	−0.0089 (17)	0.0004 (14)
C35	0.037 (2)	0.0222 (16)	0.0357 (19)	0.0012 (15)	−0.0099 (17)	0.0013 (14)
C36	0.050 (3)	0.0279 (18)	0.055 (2)	0.0076 (17)	−0.019 (2)	−0.0024 (17)
C37	0.060 (3)	0.0310 (19)	0.066 (3)	0.0086 (19)	−0.029 (2)	0.0057 (18)
C38	0.048 (3)	0.079 (3)	0.076 (3)	0.010 (2)	−0.023 (2)	0.014 (2)

### Geometric parameters (Å, °)

**Table:**

Cu1—N1^i^	1.997 (2)	C21—H21B	0.9900
Cu1—N1	1.997 (2)	C22—C23'	1.544 (4)
Cu1—N2	2.002 (2)	C22—C23	1.550 (5)
Cu1—N2^i^	2.002 (2)	C22—H22A	1.0101
N1—C1	1.379 (3)	C22—H22B	1.0014
N1—C4	1.387 (3)	C22—H22C	1.0006
N2—C9	1.371 (3)	C22—H22D	0.9822
N2—C6	1.383 (3)	C23—C24	1.537 (5)
O1—C14	1.379 (3)	C23—H23A	0.9900
O1—C17	1.414 (4)	C23—H23B	0.9900
O2—C28	1.381 (3)	C24—H24A	0.9800
O2—C31	1.436 (3)	C24—H24B	0.9800
C1—C10	1.392 (4)	C24—H24C	0.9800
C1—C2	1.439 (4)	C23'—C24'	1.540 (5)
C2—C3	1.344 (4)	C23'—H23C	0.9900
C2—H2	0.9500	C23'—H23D	0.9900
C3—C4	1.429 (4)	C24'—H24D	0.9800
C3—H3	0.9500	C24'—H24E	0.9800
C4—C5	1.393 (4)	C24'—H24F	0.9800
C5—C6	1.393 (4)	C25—C30	1.379 (4)
C5—C25	1.507 (4)	C25—C26	1.379 (4)
C6—C7	1.429 (4)	C26—C27	1.399 (4)
C7—C8	1.343 (4)	C26—H26	0.9500
C7—H7	0.9500	C27—C28	1.375 (4)
C8—C9	1.439 (4)	C27—H27	0.9500
C8—H8	0.9500	C28—C29	1.383 (4)
C9—C10^i^	1.394 (4)	C29—C30	1.387 (4)
C10—C9^i^	1.395 (4)	C29—H29	0.9500
C10—C11	1.504 (4)	C30—H30	0.9500
C11—C12	1.383 (4)	C31—C32	1.510 (4)
C11—C16	1.381 (4)	C31—H31A	0.9900
C12—C13	1.393 (4)	C31—H31B	0.9900
C12—H12	0.9500	C32—C33	1.522 (4)
C13—C14	1.381 (4)	C32—H32A	0.9900
C13—H13	0.9500	C32—H32B	0.9900
C14—C15	1.369 (4)	C33—C34	1.519 (4)
C15—C16	1.396 (4)	C33—H33A	0.9900
C15—H15	0.9500	C33—H33B	0.9900
C16—H16	0.9500	C34—C35	1.519 (4)
C17—C18	1.523 (4)	C34—H34A	0.9900
C17—H17A	0.9900	C34—H34B	0.9900
C17—H17B	0.9900	C35—C36	1.523 (4)
C18—C19	1.533 (4)	C35—H35A	0.9900
C18—H18A	0.9900	C35—H35B	0.9900
C18—H18B	0.9900	C36—C37	1.520 (4)
C19—C20	1.521 (4)	C36—H36A	0.9900
C19—H19A	0.9900	C36—H36B	0.9900
C19—H19B	0.9900	C37—C38	1.510 (5)
C20—C21	1.521 (4)	C37—H37A	0.9900
C20—H20A	0.9900	C37—H37B	0.9900
C20—H20B	0.9900	C38—H38A	0.9800
C21—C22	1.518 (4)	C38—H38B	0.9800
C21—H21A	0.9900	C38—H38C	0.9800
			
N1^i^—Cu1—N1	180.00 (6)	C23—C22—H22A	124.4
N1^i^—Cu1—N2	89.96 (9)	C21—C22—H22A	106.2
N1—Cu1—N2	90.04 (9)	C23'—C22—H22B	130.3
N1^i^—Cu1—N2^i^	90.04 (9)	C23—C22—H22B	98.8
N1—Cu1—N2^i^	89.96 (9)	C21—C22—H22B	106.9
N2—Cu1—N2^i^	180.0	H22A—C22—H22B	104.8
C1—N1—C4	105.4 (2)	C23'—C22—H22C	92.0
C1—N1—Cu1	127.47 (17)	C23—C22—H22C	125.5
C4—N1—Cu1	127.10 (19)	C21—C22—H22C	108.3
C9—N2—C6	105.7 (2)	C23'—C22—H22D	122.9
C9—N2—Cu1	126.93 (18)	C21—C22—H22D	109.4
C6—N2—Cu1	127.40 (18)	H22C—C22—H22D	107.4
C14—O1—C17	117.9 (3)	C22—C23—C24	111.1 (9)
C28—O2—C31	116.9 (2)	C22—C23—H23A	109.4
N1—C1—C10	125.6 (2)	C24—C23—H23A	109.4
N1—C1—C2	110.0 (2)	C22—C23—H23B	109.4
C10—C1—C2	124.4 (3)	C24—C23—H23B	109.4
C3—C2—C1	106.9 (3)	H23A—C23—H23B	108.0
C3—C2—H2	126.5	C24'—C23'—C22	106.8 (7)
C1—C2—H2	126.5	C24'—C23'—H23C	110.4
C2—C3—C4	107.8 (2)	C22—C23'—H23C	110.4
C2—C3—H3	126.1	C24'—C23'—H23D	110.4
C4—C3—H3	126.1	C22—C23'—H23D	110.4
C5—C4—N1	125.9 (2)	H23C—C23'—H23D	108.6
C5—C4—C3	124.2 (2)	C23'—C24'—H24D	109.5
N1—C4—C3	109.8 (2)	C23'—C24'—H24E	109.5
C6—C5—C4	123.8 (2)	H24D—C24'—H24E	109.5
C6—C5—C25	118.4 (3)	C23'—C24'—H24F	109.5
C4—C5—C25	117.8 (2)	H24D—C24'—H24F	109.5
N2—C6—C5	125.6 (3)	H24E—C24'—H24F	109.5
N2—C6—C7	109.8 (2)	C30—C25—C26	118.0 (3)
C5—C6—C7	124.4 (2)	C30—C25—C5	122.5 (3)
C8—C7—C6	107.4 (3)	C26—C25—C5	119.4 (3)
C8—C7—H7	126.3	C25—C26—C27	121.4 (3)
C6—C7—H7	126.3	C25—C26—H26	119.3
C7—C8—C9	107.1 (3)	C27—C26—H26	119.3
C7—C8—H8	126.5	C28—C27—C26	119.5 (3)
C9—C8—H8	126.5	C28—C27—H27	120.3
N2—C9—C10^i^	126.4 (3)	C26—C27—H27	120.3
N2—C9—C8	109.9 (2)	O2—C28—C27	124.4 (3)
C10^i^—C9—C8	123.7 (3)	O2—C28—C29	115.8 (3)
C1—C10—C9^i^	123.5 (3)	C27—C28—C29	119.8 (3)
C1—C10—C11	118.2 (2)	C28—C29—C30	119.7 (3)
C9^i^—C10—C11	118.4 (2)	C28—C29—H29	120.1
C12—C11—C16	118.0 (3)	C30—C29—H29	120.1
C12—C11—C10	121.7 (3)	C25—C30—C29	121.5 (3)
C16—C11—C10	120.3 (3)	C25—C30—H30	119.2
C11—C12—C13	121.1 (3)	C29—C30—H30	119.2
C11—C12—H12	119.4	O2—C31—C32	108.0 (3)
C13—C12—H12	119.4	O2—C31—H31A	110.1
C12—C13—C14	119.9 (3)	C32—C31—H31A	110.1
C12—C13—H13	120.0	O2—C31—H31B	110.1
C14—C13—H13	120.0	C32—C31—H31B	110.1
C15—C14—O1	124.9 (3)	H31A—C31—H31B	108.4
C15—C14—C13	119.7 (3)	C31—C32—C33	112.4 (2)
O1—C14—C13	115.4 (3)	C31—C32—H32A	109.1
C14—C15—C16	120.0 (3)	C33—C32—H32A	109.1
C14—C15—H15	120.0	C31—C32—H32B	109.1
C16—C15—H15	120.0	C33—C32—H32B	109.1
C11—C16—C15	121.3 (3)	H32A—C32—H32B	107.9
C11—C16—H16	119.4	C32—C33—C34	115.0 (2)
C15—C16—H16	119.4	C32—C33—H33A	108.5
O1—C17—C18	107.3 (3)	C34—C33—H33A	108.5
O1—C17—H17A	110.2	C32—C33—H33B	108.5
C18—C17—H17A	110.2	C34—C33—H33B	108.5
O1—C17—H17B	110.2	H33A—C33—H33B	107.5
C18—C17—H17B	110.2	C35—C34—C33	111.9 (2)
H17A—C17—H17B	108.5	C35—C34—H34A	109.2
C17—C18—C19	112.4 (3)	C33—C34—H34A	109.2
C17—C18—H18A	109.1	C35—C34—H34B	109.2
C19—C18—H18A	109.1	C33—C34—H34B	109.2
C17—C18—H18B	109.1	H34A—C34—H34B	107.9
C19—C18—H18B	109.1	C34—C35—C36	115.5 (2)
H18A—C18—H18B	107.9	C34—C35—H35A	108.4
C20—C19—C18	114.1 (2)	C36—C35—H35A	108.4
C20—C19—H19A	108.7	C34—C35—H35B	108.4
C18—C19—H19A	108.7	C36—C35—H35B	108.4
C20—C19—H19B	108.7	H35A—C35—H35B	107.5
C18—C19—H19B	108.7	C37—C36—C35	113.3 (3)
H19A—C19—H19B	107.6	C37—C36—H36A	108.9
C19—C20—C21	111.7 (3)	C35—C36—H36A	108.9
C19—C20—H20A	109.3	C37—C36—H36B	108.9
C21—C20—H20A	109.3	C35—C36—H36B	108.9
C19—C20—H20B	109.3	H36A—C36—H36B	107.7
C21—C20—H20B	109.3	C36—C37—C38	114.0 (3)
H20A—C20—H20B	107.9	C36—C37—H37A	108.8
C20—C21—C22	115.5 (3)	C38—C37—H37A	108.8
C20—C21—H21A	108.4	C36—C37—H37B	108.8
C22—C21—H21A	108.4	C38—C37—H37B	108.8
C20—C21—H21B	108.4	H37A—C37—H37B	107.7
C22—C21—H21B	108.4	C37—C38—H38A	109.5
H21A—C21—H21B	107.5	C37—C38—H38B	109.5
C23'—C22—C23	39.6 (4)	H38A—C38—H38B	109.5
C23'—C22—C21	114.4 (4)	C37—C38—H38C	109.5
C23—C22—C21	113.8 (4)	H38A—C38—H38C	109.5
C23'—C22—H22A	89.5	H38B—C38—H38C	109.5

Symmetry codes: (i) −*x*, −*y*, −*z*.

## References

[bb1] Anderson, H. L. & Sanders, J. K. M. (1995). *J. Chem. Soc. Perkin Trans. 1*, pp. 2223–2229.

[bb2] Bruker (2002). *SMART* and *SAINT* Bruker AXS Inc., Madison, Wisconsin, USA.

[bb3] Fleischer, E. B. (1963). *J. Am. Chem. Soc.* **85**, 1353–1354.

[bb4] Fleischer, E. B., Miller, C. K. & Webb, L. E. (1964). *J. Am. Chem. Soc.* **86**, 2342–2347.

[bb5] Gunter, M. J. & Johnston, M. R. (1992). *J. Chem. Soc. Chem. Commun.* **17**, 1163–1165.

[bb6] Gust, D. & Moore, T. A. (1985). *J. Photochem.* **29**, 174–184.

[bb7] He, H.-S. (2007). *Acta Cryst.* E**63**, m976–m977.

[bb8] Holten, D., Bocian, D. F. & Lindsey, J. S. (2002). *Acc. Chem. Res.* **35**, 57–69. 10.1021/ar970264z 11790089

[bb9] Sheldrick, G. M. (2004). *SADABS* University of Göttingen, Germany.

[bb10] Sheldrick, G. M. (2008). *Acta Cryst.* A**64**, 112–122. 10.1107/S0108767307043930 18156677

